# Tailored Practical Management of Patients With Atrial Fibrillation: A Risk Factor-Based Approach

**DOI:** 10.3389/fcvm.2019.00017

**Published:** 2019-03-12

**Authors:** Daniele Pastori, Danilo Menichelli, Rony Gingis, Pasquale Pignatelli, Francesco Violi

**Affiliations:** I Clinica Medica, Atherothrombosis Centre, Department of Internal Medicine and Medical Specialties of Sapienza University, Rome, Italy

**Keywords:** atrial fibrillation, practical management, NOAC, bleeding, scores, ABC

## Abstract

The management of antithrombotic therapy for thromboprophylaxis in patients with atrial fibrillation (AF) has been recently evolved by the progressive replacement of vitamin K antagonists with the non-vitamin K antagonist oral anticoagulants (NOACs). However, while these drugs are effective in reducing ischemic stroke/systemic embolism, a still high rate of cardiovascular events is present in the AF population. A tailored integrated approach to patients with AF is therefore necessary to reduce both thromboembolic events and cardiovascular disease. This approach should consist in the assessment of individual risk factors for ischemic and bleeding events in order to choose the most appropriate anticoagulant treatment according to patient's characteristics and preference. To this purpose, several risk scores have been developed and validated to stratify thromboembolic and hemorrhagic risk. This review provides an individual-based strategy for the management of patients with AF, from a risk-factor based approach to a tailored prescription and monitoring of NOACs. In particular, we reported an updated practical management strategy for AF patients in specific clinical situations such as those (1) experiencing a major bleeding, (2) requiring a switch to another antithrombotic regimen, (3) restarting anticoagulation after acute ischemic stroke, (4) suffering from an acute coronary artery disease (acute coronary syndrome or undergoing cardiac revascularization).

## Introduction

Atrial fibrillation (AF) is the most common heart rhythm disorder, responsible for approximately one-third of hospitalizations for cardiac rhythm disturbances in the United States of America (USA) (1). The prevalence and incidence of AF are increasing, and AF is predicted to affect 6–12 million people in the USA by 2050 and 17.9 million in Europe by 2060, and hence determine an impact on healthcare costs (1). AF is associated with an increased morbidity and mortality, due to risk of ischemic stroke, systemic embolism, heart failure, and cognitive impairment, overall reducing the quality and quantity of life in these patients (2). Thus, AF has been well-recognized as a risk factor for thromboembolic stroke, increasing its incidence by 4- to 5-fold (3). Furthermore, it has been shown that up to 30% of stroke of undetermined source may be attributable to AF (4).

A growing body of evidence suggest that, in addition to thromboembolism, the natural history of AF is complicated by a high rate of cardiovascular events (5, 6), with 7 in 10 deaths being cardiovascular-related (7).

Oral anticoagulation (OAC) with Vitamin K antagonist (VKAs) has represented the mainstay of thromboprophylaxis in patients with AF over the last decades. Yet, VKAs therapy has some clinical challenges due to the need for close monitoring of INR, drug interactions and a narrow therapeutic range.

All these issues have led to the development of the non-vitamin K antagonist oral anticoagulants (NOACs), including a factor IIa inhibitor (dabigatran) and factor Xa inhibitors (apixaban, edoxaban, rivaroxaban). In phase 3 clinical trials, NOACs have been shown to be as effective as VKAs for the prevention of ischemic stroke, with a safer profile, mostly related to a significant reduction of the rate of intracranial hemorrhage (ICH) (8, 9).

The introduction of NOACs increased the number of therapeutic tools for clinicians in the management of patients with AF. However, given the different characteristics of these drugs, a careful evaluation of patient's characteristics and comorbidities is needed to identify the most appropriate antithrombotic regimen according to patient profile. Identification of risk factors for ischemic and bleeding events, especially the modifiable ones should guide the choice of the anticoagulant drug.

The aim of this review is to provide a summary of current evidence on risk stratification strategies for patients with AF and to provide an updated practical approach to guide the management of anticoagulation therapy in specific situations.

## Stroke and Bleeding Risk Stratification

Several scoring systems have been developed to assess the risk of ischemic and hemorrhagic events. Table 1 reports a summary of the most studied risk scores for thromboembolic (Panel A) and hemorrhagic (Panel B) risk.

**Table 1:** Summary of thromboembolic (Panel A) and bleeding (Panel B) risk scores.

**CHADS_**2**_(10)**	**CHA_**2**_DS_**2**_-VASc (11)**	**ABC stroke (12)**	**ATRIA (13) without prior stroke**	**ATRIA (13) with prior stroke**
**A. THROMBOEMBOLIC RISK SCORES**				
Congestive heart failure (1 point)	Congestive heart failure (1 point)	Age (44–90 years)	Age (years)	Age (years)
			≥85 (6 points)	≥85 (9 points)
			75–84 (5 points)	75–84 (7 points)
			65–74 (3 points)	65–74 (7 points)
			<65 (0 points)	<65 (8 points)
Hypertension (1 point)	Hypertension (1 point)	Biomarkers (Troponin I and NT-proBNP)	Female sex (1 point)	Female sex (1 point)
Age ≥75 years (1 point)	Age ≥75 years (2 points)	Clinical history of stroke/TIA	Diabetes (1 point)	Diabetes (1 point)
Diabetes (1 point)	Diabetes (1 point)		Congestive heart failure (1 point)	Congestive heart failure (1 point)
Previous stroke/TIA (2 points)	Previous Stroke/TIA (2 points)		Hypertension (1 point)	Hypertension (1 point)
	Vascular disease^*^ (1 point)		Proteinuria (1 point)	Proteinuria (1 point)
	Age (65–74 years) (1 point)		eGFR < 45 ml/min or ESRD (1 point)	eGFR < 45 ml/min or ESRD (1 point)
	Female sex (1 point)			
**HAS-BLED (****14****)**	**ATRIA (****15****)**	**HEMORR**_**2**_**HAGES (****16****)**	**ORBIT-AF (****17****)**	**ABC bleeding (****18****)**
**B. HEMORRHAGIC RISK SCORES**				
Hypertension (uncontrolled) (1 point)	Anemia (3 points)	Hepatic/renal disease (1 point)	Age ≥75 years (1 point)	Age
Abnormal renal/ liver function (1 point)	eGFR < 30 ml/min or Dialysis (3 points)	Alcohol abuse (1 point)	Reduced hemoglobin/ haematocrit/ history of anemia (2 points) ^**^	Biomarkers^***^
Stroke (1 point)	Age ≥75 years (2 points)	Malignancy (1 point)	Bleeding history (2 points)	Clinical history of bleeding
Bleeding history (1 point)	Prior hemorrhage (1 point)	Older ≥75 years (1 point)	eGFR <60 ml/min/1.73m^2^ (1 point)	
Labile INR (1 point)	Hypertension (1 point)	Reduced platelet count <75,000 or antiplatelet therapy (1 point)	Antiplatelet drug use (1 point)	
Elderly > 65 years (1 point)		Re-bleeding risk (2 points)		
Drugs/alcohol use (1 point)		Hypertension (uncontrolled) (1 point)		
		Anemia (1 point)		
		Genetic factors (CYP2C9^*^2, CYP2C9^*^3) (1 point)		
		Excessive fall risk (neuropsychiatric disease) (1 point)		
		Stroke (1 point)		

* *Peripheral artery disease, myocardial infarction, aortic plaque*.

** *< 13mg/dL in men and < 12 mg/dL in women; haematocrit (<40% in men and <36% in women)*.

*** *Including growth differentiation factor-15 (GDF-15), Troponin T (cTnT-hs) and hemoglobin*.

*eGFR, estimated glomerular filtration; ESRD, end-stage renal disease; INR, international normalized ratio*.

### Thromboembolic Risk Stratification

Among clinical scores for thromboembolic risk (Table 1A), the first approved score for cardioembolic stroke risk stratification has been the CHADS_2_ score (10) which classified patients into three groups as low (i.e., 0 point), moderate (i.e., 1–2 points), high (i.e., 3–6 points) risk for stroke. All patients with a CHADS_2_ score ≥2 were candidate to receive an anticoagulation treatment (19).

The CHADS_2_ score were then refined into the new CHA_2_DS_2_-VASc score (11) (Table 1A), which has represented a step forward a personalized risk stratification for patients with AF.

The greatest advantage of CHA_2_DS_2_ VASc score over CHADS_2_ score is to identify truly low-risk patients for ischemic stroke who are unlikely to benefit from OAC treatment (i.e., CHA_2_DS_2_ -VASc score 0–1) (11).

Recent evidence showed that patients with one additional risk factor beyond sex are at increased risk of thromboembolic events, suggesting that OAC should probably be considered also in this subgroup of patients (20).

The new 2019 AHA guidelines recommend that men and women with AF but no additional risk factors should not be prescribed on OAC and in patients with one additional risk factor beyond sex, prescribing an oral anticoagulant to reduce thromboembolic stroke risk may be considered (21).

Finally, the ATRIA score was validated in a cohort of 10,927 AF patients and externally validated in a community-based cohort of 33,247 AF patients (13) (Table 1A).

#### Recent Studies Compared the Predictive Value of These Scores

The ATRIA, CHADS_2_, and CHA_2_DS_2_-VASc score were compared (22) in a population of 60,594 patients in whom the annualized stroke rate was 2.99%. The C-statistics (95% confidence interval) were 0.70 (0.69–0.71) for the ATRIA, 0.68 (0.67–0.69) for CHADS_2_, and 0.68 (0.67–0.69) for CHA_2_DS_2_-VASc risk score. Furthermore, the ATRIA score had a net reclassification improvement of 0.23 (0.22–0.25) compared with CHA_2_DS_2_-VASc. This study shows that ATRIA score performed better than CHADS_2_ and CHA_2_DS_2_-VASc score, mostly in the identification of low-risk patients.

The ABC (Age, Biomarkers, Clinical history) stroke risk score (12) (Table 1A) is a biomarker-based score derived from the cohort of 14,701 AF patients from the ARISTOTLE trial. External validation was performed in an independent cohort of 1,400 AF patients. Each item scores 0–10 point according to nomogram reference values and the sum of these points gives the 1 and 3-year risk of ischemic stroke. This score identifies three risk classes: low (<1%), medium (1–2%), and high (>2%) risk. The ABC-stroke score achieved better prediction than the CHA_2_DS_2_-VASc score in both internal (c-index 0.68 vs. 0.62, *p* < 0.001, respectively) and external cohort (0.66 vs. 0.58, *p* < 0.001, respectively) (12).

In the cohort of the RE-LY trial that included 8,356 anticoagulated patients with AF (23), the ABC-stroke score performed better than both the CHA_2_DS_2_VASc and ATRIA stroke scores (c-statistics of 0.65, 0.60, and 0.61, respectively).

Currently, the ESC 2016 (24) and 2019 AHA guidelines (21) recommend using the CHA_2_DS_2_-VASc to assess thromboembolic risk of AF patients, as this score has the most consolidated evidence and includes simple variables to be calculated.

### Bleeding Risk Stratification

European guidelines recommend to stratify bleeding risk before the prescription of an anticoagulant drug, aiming to identify potentially modifiable risk factors (24). High bleeding risk score should not represent an absolute contraindication to OAC, but it claims a closer monitoring of patients starting OAC (24). The bleeding risk scores available so far include the HAS-BLED, ATRIA, HEMORR_2_HAGES, ABC, and ORBIT score, summarized in Table 1B.

The HAS BLED score (14) (Table 1B) was developed to predict the risk of major bleeding (ICH, hospitalization, hemoglobin decrease > 2 g/L, and/or transfusion) in a real-world AF population of 3,978 patients with 55 bleeding events at 1 year of follow-up, showing a good predictive ability (C statistic 0.72). An HAS BLED score ≥3 identifies a high risk of a major bleeding.

The ATRIA bleeding score was developed on 9,186 AF patients suffering 461 major hemorrhages (1.4%/year) (15). The score included five variables for a total of 10 points (Table 1B). Patients were divided in “low” (≤3 points), “intermediate” (4 points), and “high” (5–10 points) bleeding risk. The c-index for the continuous risk score was 0.74 (95%CI 0.70–0.78) (15).

The HEMORR_2_HAGES (16) (Table 1B) score was based on 3,791 Medicare beneficiaries with AF with a bleeding rate of 5.2 per 100 patient-years. The score has a global modest predictive accuracy (c statistic of 0.67), with a bleeding rate increasing up to 12.3 per 100 patient-years in patients with ≥5 points (16).

A metanalysis by Caldeira et al. (25) compared the three bleeding risk scores showing that HAS BLED had a better sensitivity than HEMORR_2_HAGES and ATRIA, along with a worse diagnostic Odds Ratio compared with HEMORR_2_HAGES (2.1 vs. 2.9, respectively) and better compared with ATRIA (2.22 vs. 1.98, respectively). The author concluded that HAS BLED should be preferred in assessing the risk of bleeding in AF patients, given its simplicity and greater sensitivity compared to other scores (25).

The Outcomes of the Registry for Better Informed Treatment of Atrial Fibrillation (ORBIT) bleeding score (17) is composed of five items (Table 1B). The score was derived on 7,411AF patients from the ORBIT cohort and tested on 14,264 patients from the ROCKET-AF trial. In the ORBIT registry, the ORBIT score showed a c-index of 0.67 (95%CI 0.64–0.69), higher than the HAS BLED (c-index0.64, 95%CI 0.62–0.67) and similar to the ATRIA bleeding score (c-index 0.66, 95%CI 0.63–0.68). Similar results were found in the external cohort of the ROCKET-AF (c-indexes 0.62, 0.59, 0.60, respectively) (17).

Finally, the ABC (age, biomarkers, clinical history) bleeding risk score is a biomarker-based scheme (Table 1B) (18). The internal validation was performed on 14,537 AF patients from the ARISTOTLE trial, that randomized AF patients to receive Apixaban or VKAs treatment, and on 8,468 AF patients from RE-LY trial. ABC score performed better than HAS-BLED and ORBIT scores for major bleeding in both derivation (c-index was 0.68, 0.61, and 0.65, respectively) and validation cohort (c-index 0.71, 0.62, and 0.68, respectively (18).

None of the above-mentioned scores have showed a high enough accuracy to be recommended as the gold standard for bleeding risk stratification in AF patients. Therefore, current guidelines advise to assess known factors that may increase bleeding risk. Overall, these factors can be divided into modifiable (e.g., concurrent non-steroidal anti-inflammatory and antiplatelet drugs, alcohol use, uncontrolled hypertension) and not modifiable (e.g., age, previous major hemorrhage) (24).

### Composite Endpoints: TIMI-AF and 2MACE

Independently from the type of complication (ischemic or hemorrhagic), any clinically relevant event occurring to patients with AF has a negative impact on patient's personal history and prognosis, leading to a high rate of OAC discontinuation (26) and subsequent high risk of recurrent event and mortality (27). This have led to the development of the composite endpoints concept, such as Major Adverse Cardiovascular Events (MACEs) and Net Clinical Outcomes (NCOs), as a measure of the global risk of AF patients of experiencing a clinical event during lifetime.

Two scores that evaluated composite endpoints in AF have been developed, namely 2MACE and TIMI-AF score (28).

The 2MACE score (29) included five items, Metabolic Syndrome (2 points), Age ≥75 (2 points), MI/revascularization (1 point), Congestive heart failure (1 point), and stroke/TIA (1 point) scoring 0–7 points. It was developed to predict MACEs, defined by a fatal/non-fatal MI, cardiac revascularization, and cardiovascular death. Patients with a 2MACE score ≥3 were classified at high risk for MACE (29). This score was developed on a cohort of 1,019 AF patients, and externally validated on 1,089 AF patients. In this study, 111 MACE events occurred in the internal cohort and 68 in the external cohort. The 2MACE showed a good predictive capacity with c-index of 0.79 in the internal and 0.66 in the external cohort (29).

The 2MACe score has recently received two additional independent external validations (30, 31). In a cohort of *n* = 794 AF patients without CAD the 2MACE score showed a good predictive ability for MACE (C-statistic, 0.699; 95%CI, 0.648–0.750; *p* < 0.001) (30).

Furthermore, the 2MACE has been tested also in the Spanish FANTASIIA registry and Murcia cohort, confirming that patients with a 2MACE score ≥3 had a significantly higher incidence of MACE as compared to those with a score <3 (1.94 vs. 0.81%/year in the Murcia cohort and 1.71 vs. 6.01%/year in the FANTASIIA registry, respectively) (31).

The TIMI-AF score (32) was developed on the warfarin arm of ENGAGE AF-TIMI 48 trial with 2,898 patients. TIMI-AF is composed of 17 items and was developed to predict NCO, including disabling stroke, life-threatening bleeding, and all-cause mortality. In a median of 2.7 years, 457 NCO events occurred (6.05%/year). TIMI-AF score had a c-statistics value of 0.693, but it has not been externally validated (32).

Rivera-Caravaca et al. (33) compared the TIMI-AF with CHA_2_DS_2_-VASc and HAS-BLED scores on 1,156 AF patients with 563 NCOs during a 6.5 years follow-up (6.07%/year). The TIMI-AF predictive performance didn't differ from CHA_2_DS_2_-VASc and HAS-BLED (0.678 vs. 0.677 and 0.644 vs. 0.671, respectively). The study concluded that TIMI-AF was not superior to CHA_2_DS_2_-VASc or HAS-BLED (33).

The 2MACE and TIMI-AF scores were compared in a “real world” cohort of 907 AF patients and in a cohort of 2,265 patients from the AMADEUS trial (34). Endpoints of the cohorts were MACE, NCO and Clinically Relevant Events (CREs, a combination of MACE and NCO). The scores showed similar predictive value for all composite endpoints, with the advantage of the 2MACE of being easier to calculate in a daily clinical practice (34).

## Factors Affecting the Choice of Anticoagulant Therapy

Clinical and biochemical factors may affect the choice of OAC, including the presence of valvular heart disease (VHD), renal function, the quality of OAC in patients already taking VKAs, drug interactions, patient's needs and preference (Table 2).

**Table 2:** Factors to be evaluated for the switching to non-vitamin K antagonist oral anticoagulants.

**Patients already on OAC**	**Patients starting OAC**
TiTR < 65–70% during last 6–12 months (35, 36)	TtTR >18 days when starting VKA therapy (37)
Previous thromboembolic event under well-controlled VKA therapy/ other NOAC (38)	Unable to undergo frequent INR check (21)
	SAMe-TT_2_R_2_ ≥2 (39)
**COMMON FACTORS**	
Renal function (40)	Drug interactions (40)
Elderly (≥ 75 years) (41–44)	Patient's preference (45)
History of intracranial hemorrhage (38)	Concomitant antiplatelet drugs (38)
Presence and type of valvular heart disease (46)	

*OAC, oral anticoagulation; TiTR, time in therapeutic range, TtTR, time to therapeutic range, VKA, vitamin K antagonist*.

For AF patients already on VKAs, several studies investigated the quality of anticoagulation, as assessed by the time in therapeutic range (TiTR), which reflects the time spent within the range of INR (47). While well-managed VKA therapy (i.e., TiTR ≥65–70%) still represents a valid option for stroke prevention in patients with AF (48), a low TiTR is associated with increased thromboembolism, cardiovascular events (CVEs) (35), mortality and bleeding (36). Thus, patients with low TiTR would benefit from switching to NOACs.

Another issue is represented by the variation of TiTR over time. A previous study showed that a decline of TiTR from above to below 70% can be observed in at least 20% of AF patients, and that patients with worsening TiTR had a similar risk of CVEs compared to patients with a constantly low TiTR (49). In a recent study on 4,772 AF patients from Danish National registry, the proportion of AF patients with worsening TiTR was even higher, as only 55.7% out of 1,691 AF patients with TiTR ≥70%, maintained a high TiTR after 12 months of follow-up (50).

These findings suggest that when a patient experience a reduction of TiTR, the risk of adverse outcomes significantly increases and switching to NOACs may be particularly beneficial.

For patients starting OAC, the quality of anticoagulation therapy can be predicted by clinical risk scores, summarized in Table 3. One of the most studied is the SAMe-TT_2_R_2_ score that showed a good discrimination performance in internal validation (c-index 0.72) and external validation (c-index 0.70) cohorts (39). Score of 0–1 point predicts a good TiTR, conversely if SAMe-TT_2_R_2_ score is ≥ 2, VKAs would not be optimal (39). Recently, a review by Zulkifly et al. (47) which included 19 studies investigating the predictive ability of SAMe-TT_2_R_2_ score in patients with AF or venous thromboembolism (VTE), confirmed the usefulness of this score in predicting good anticoagulation (47).

**Table 3:** Scores for the prediction of anticoagulation quality with vitamin K antagonists.

**Variables**	**SAMe-TT_**2**_R_**2**_ (39) (points)**	**PROSPER (51) (points)**	Geisinger model (52)^*^
1	Sex female (1)	Pneumonia (1)	Alcohol abuse
2	Age < 60 year (1)	Renal dysfunction (2)	Anemia
3	Medical history (1)	Oozing blood (1)	Lung disease
4	Treatment (1)	Staying in hospital (1)	Hemorrhagic stroke
5	Tobacco use (2)	Pain medications (1)	Thrombocytopenia
6	Race (2)	No enhanced anticoagulation care (4)	Venous thromboembolism
7		Rx for antibiotics (1)	Antiarrhythmic drugs
8			Aspirin use
9			Red blood cells count
10			Red blood cells distribution
11			Neutrophil %
12			Albumin, g/dL
13			Body mass index
14			Systolic blood pressure
15			Age

* *If are present ≥ 4 poor TiTR factors the estimated TiTR are < 60%, if ≥ 7 poor factors, the estimated TiTR are < 50%*.

For the elderly population (≥65 years), the PROSPER (51) score was recently proposed (Table 3). All items of the score, except for the lack of dedicated healthcare structure, should be assessed with regards to a period of 6 months prior to initiating a VKA. This score, showed a better performance in predicting the TiTR>70%, thromboembolic events and hemorrhagic events compared to SAMe-TT_2_R_2_ score in this cohort of elderly patients (51). However, this score needs an external validation.

Another score based on 15 items, namely the Geisinger Model, has been recently developed on a population of 7,877 AF patients (Table 3). This score, in comparison to SAMe-TT_2_R_2_ showed better predictive performance (*R*^2^ = 3.0%) (52). However, its validation and application in clinical practice seems difficult, given the large number of variables needed to be calculated.

These scores may turn useful when evaluating patients starting OAC. For instance, patients with a high score (i.e., SAMe-TT_2_R_2_ ≥2) should be started directly on NOAC without any attempt with VKAs.

Another factor influencing the choice of OAC is the time to therapeutic range (TtTR) that is the time necessary to reach the therapeutic INR after the first administration of VKA.

The TtTR was firstly investigated in the ENSURE-AF trial, in which the TtTR was marginally correlated to cardiovascular events (53). Recently, in a prospective observational study including 1,406 AF patients followed for a mean of 31.3 months, a high TtTR (>18 days) was associated to a lower TiTR over time (OR for TiTR < 60% 1.357, 95%CI 1.056–1.745, *p* = 0.017) (37). Indeed, those patients showed an increased long-term risk of CVEs (HR: 1.857, 95% CI 1.078–3.201, *p* = 0.026) (37).

## Prescription and Follow-up of OAC: an Integrated Approach

After appropriate risk stratification, contraindications to the use of NOACs must be evaluated. In addition to absolute contraindications to OAC, such as the presence of active bleeding or severe anemia, NOACs cannot be used in specific situations, based on findings from clinical trials.

For instance, NOACs are contraindicated in patients with mechanical prosthetic valve or moderate to severe mitral stenosis (recently re-defined as EHRA type 1), while they can be prescribed to patients with biologic prosthetic heart valves or any other valvulopathy (EHRA type 2) (46).

Renal function is a major determinant in the choice of the type and dose of OAC. There are insufficient data to establish with certainty the safety of NOAC in patients with ≤15 ml/min filtrate or on dialysis, therefore their use in clinical practice in these patients should be avoided (40).

However, the 2019 AHA guidelines suggest that in patients with Creatinine Clearance <15 ml/min or on dialysis, it is reasonable to prescribe VKAs or reduced Apixaban (21).

Dabigatran is contraindicated if CrCl <30 ml/min and a dose reduction should be considered in patients with CrCl between 50 and 30 ml / min, while other NOACs can be used, with dose reduction, for CrCl <30 ml/min (54). Edoxaban should be used with caution in patients with CrCl > 95 ml / min due to a possible reduction in efficacy compared to warfarin due to over-filtration (40, 54).

Less clear is the use of NOACs in patients with chronic liver disease (CLD). All NOACs cannot be used in patients with liver cirrhosis Child-Pugh C, as these patients were not included in clinical trials. Few studies included patients with liver cirrhosis treated with NOACs for thrombosis or AF, showed that NOACs can be used without dose reduction in liver cirrhosis Child-Pugh A (55).

Moreover, Dabigatran, Apixaban and Edoxaban, but not Rivaroxaban, could be used with caution in patients with liver cirrhosis Child-Pugh B (40).

Recently, a prospective observational study on 2,330 AF patients, 1,033 on NOACs and 1,297 on VKAs, evaluated the safety and efficacy of NOACs in patients with and without CLD, defined by the non-invasive index of advanced liver fibrosis, namely FIB-4 (i.e., >3.25) (56, 57). During a mean follow up of 33.6 months, 357 bleeding events occurred. Of these, 261 in the VKA (7.2%/year) and 96 (6.4%/year) in the NOAC group (56). Patients with CLD on VKAs experienced a higher rate of major bleeding (14.3 vs. 5.6%, log-rank test *p* < 0.001) as compared to those on NOACs (5.8 vs. 9.5%, log-rank test *p* = 0.374) group (56). Furthermore, in the NOACs group no significative difference was found in CVEs incidence between patients with and without CLD (56).

These preliminary data suggested a safer profile of NOACs in AF patients with CLD, but larger studies are needed to confirm these findings.

**Figure 1:**
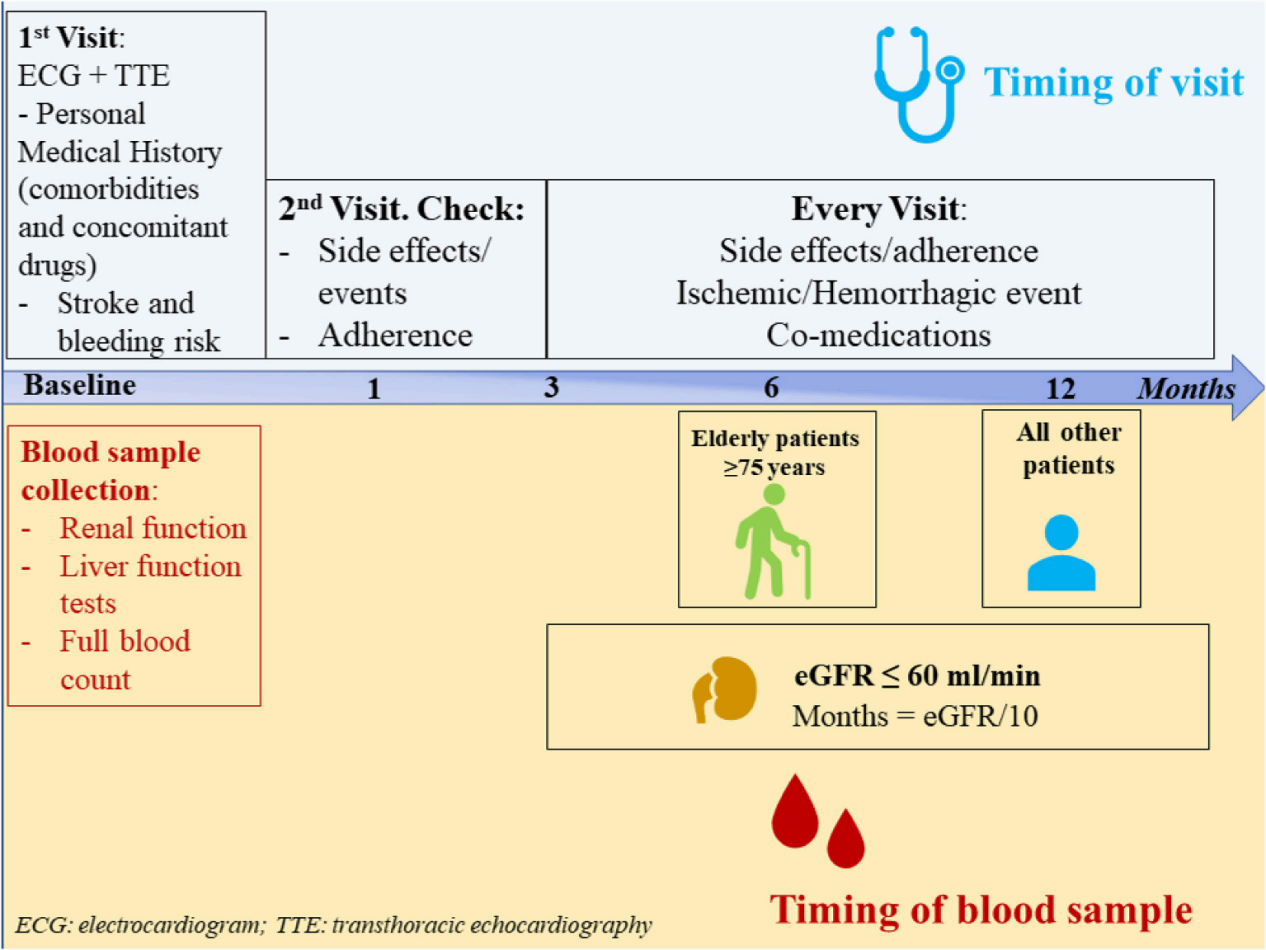
Follow-up schedule for atrial fibrillation patients prescribed on non-vitamin K antagonist oral anticoagulants.

After prescription, patients should undergo a structured follow-up. A first visit should be established after 1 month and then every 1–6 months according to patient's comorbidities and kidney function. An example of follow up chart is showed in Figure 1.

Blood sample collection is advised at baseline and then on annual basis in all patients; however, patients aged ≥ 75 years should be evaluated every 6 months, and in patients with renal impairment (eGFR ≤60 ml/min), the follow-up intervals could be generally determined by the formula: eGFR/10 (40).

During follow-up, an optimal management can improve the prognosis of patients with AF. Recently, (58) the Atrial fibrillation Better Care (ABC) pathway has been proposed as a simple integrated approach to the management of patients with AF.

The ABC pathway includes “A” Avoid stroke with Anticoagulation (i.e., well-managed VKAs therapy with TiTR >65–70%, or adherence to NOAC therapy); “B” Better symptom management, with rate or rhythm control, eventually evaluated by the EHRA score; “C” Cardiovascular risk and comorbidity management, including lifestyle factors (58). ABC pathway compliance has been associated with a reduced rate of CVEs compared to non-ABC compliant patients, when evaluated in *post-hoc* ancillary analysis of the AFFIRM trial (59), and in a real-world observational cohort ATHERO-AF study (60). In this study, ABC-compliant patients had a clear benefit in terms of lower CVEs (HR 0.439, 95%CI 0.241–0.800, *p* = 0.007) as compared to those with at least one uncontrolled risk factor. In the same cohort, adherence to ABC pathway resulted in lower healthcare-related costs (61).

Thus, management of patients with AF should not be limited to stroke prevention, but a global evaluation of patient's characteristics and risk factors is needed at baseline and during follow-up to optimize prevention strategy in these patients.

## Management of OAC in Specific Clinical Settings

### Switching Among Anticoagulants

Switching to different anticoagulant drugs is a frequent situation in daily clinical practice, as patients may experience side effects/complications during an OAC treatment, starting/stopping interfering drugs, or a worsening of clinical condition (including onset of kidney/liver disease). Thus, clinician should be aware on how to manage the switching among VKA, NOACs, low molecular weight heparin (LMWH) and intravenous unfractionated heparin (UFH), in order to guarantee an adequate antithrombotic prophylaxis and to reduce bleeding complications.

**Figure 2:**
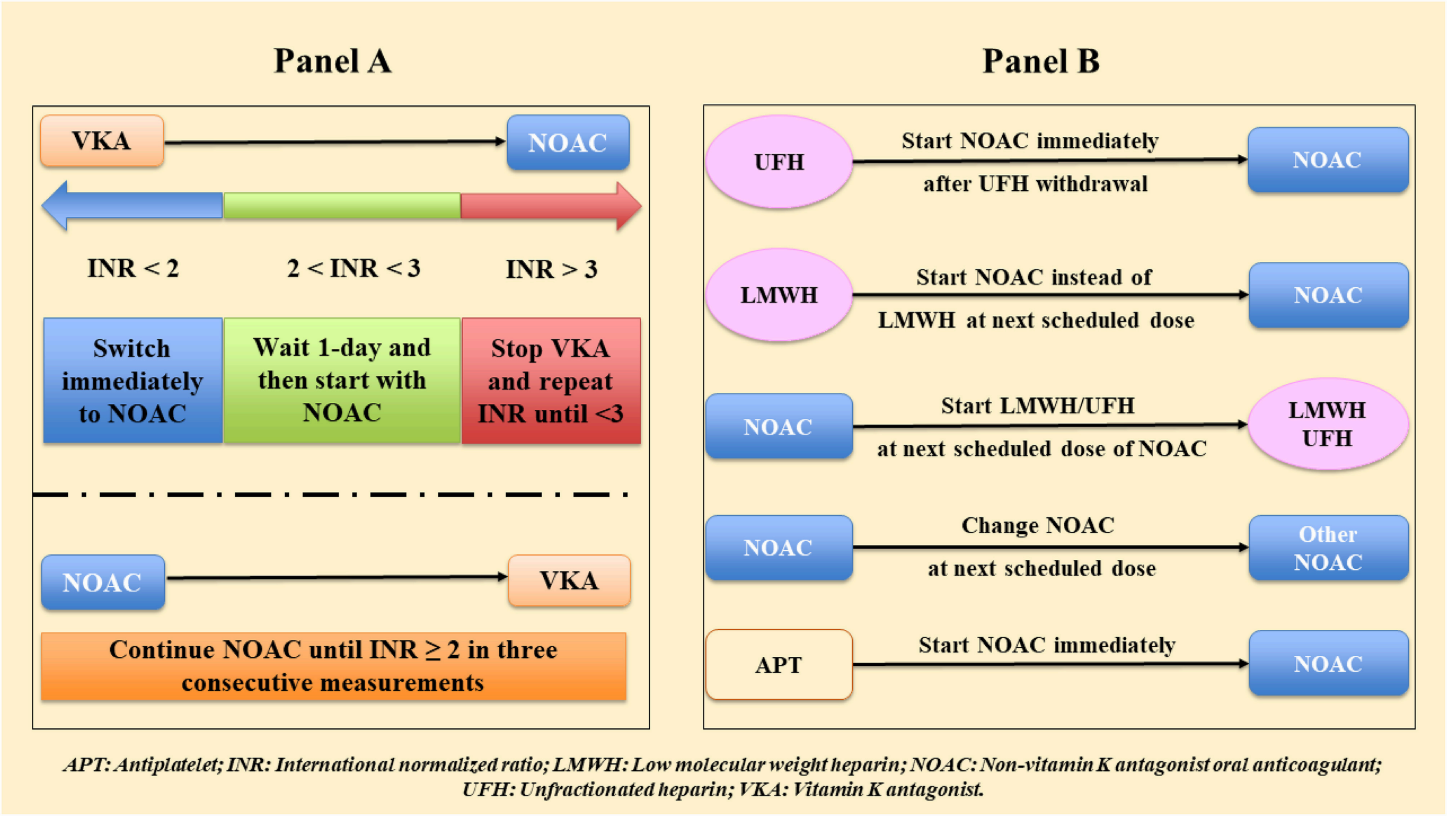
Switching among anticoagulants **(A)** between non-vitamin K antagonist oral anticoagulants (NOACs) and vitamin K antagonists (VKAs), **(B)** between NOAC and other antithrombotic regimens.

Figure 2A reports a simple scheme of switching between VKA and NOACs and backward, and Figure 2B between NOACs and other antithrombotic regimens (UFH, LMWH, antiplatelet).

### Major Bleeding

Bleeding is the most important adverse effect of OAC. The phase III clinical trials showed a major advantage of NOACs in comparison to VKAs, with a significant reduction of major hemorrhages, in particular ICH (9). Of note, an increased risk of gastrointestinal bleeding has been described with NOACs (9). Therefore, bleeding management in patients in treatment with NOACs is very important. It is helpful to distinguish between major/life-threatening and minor bleeding.

Major bleeding is defined as “*all bleeds associated with hemodynamic compromise, occurring in an anatomically critical site, or associated with a decrease of hemoglobin* ≥*2 g/dL (when baseline is known) or requiring transfusion of* ≥*2 U of packed red blood cells (RBCs)*” (62). Anatomically critical sites for major bleedings are ICH, pericardial tamponade, hemothorax, intraabdominal bleeding, retroperitoneal hematoma, extremity bleeds, and airway hemorrhages (63). Minor bleeding is defined as all bleeds not classified as major.

**Figure 3:**
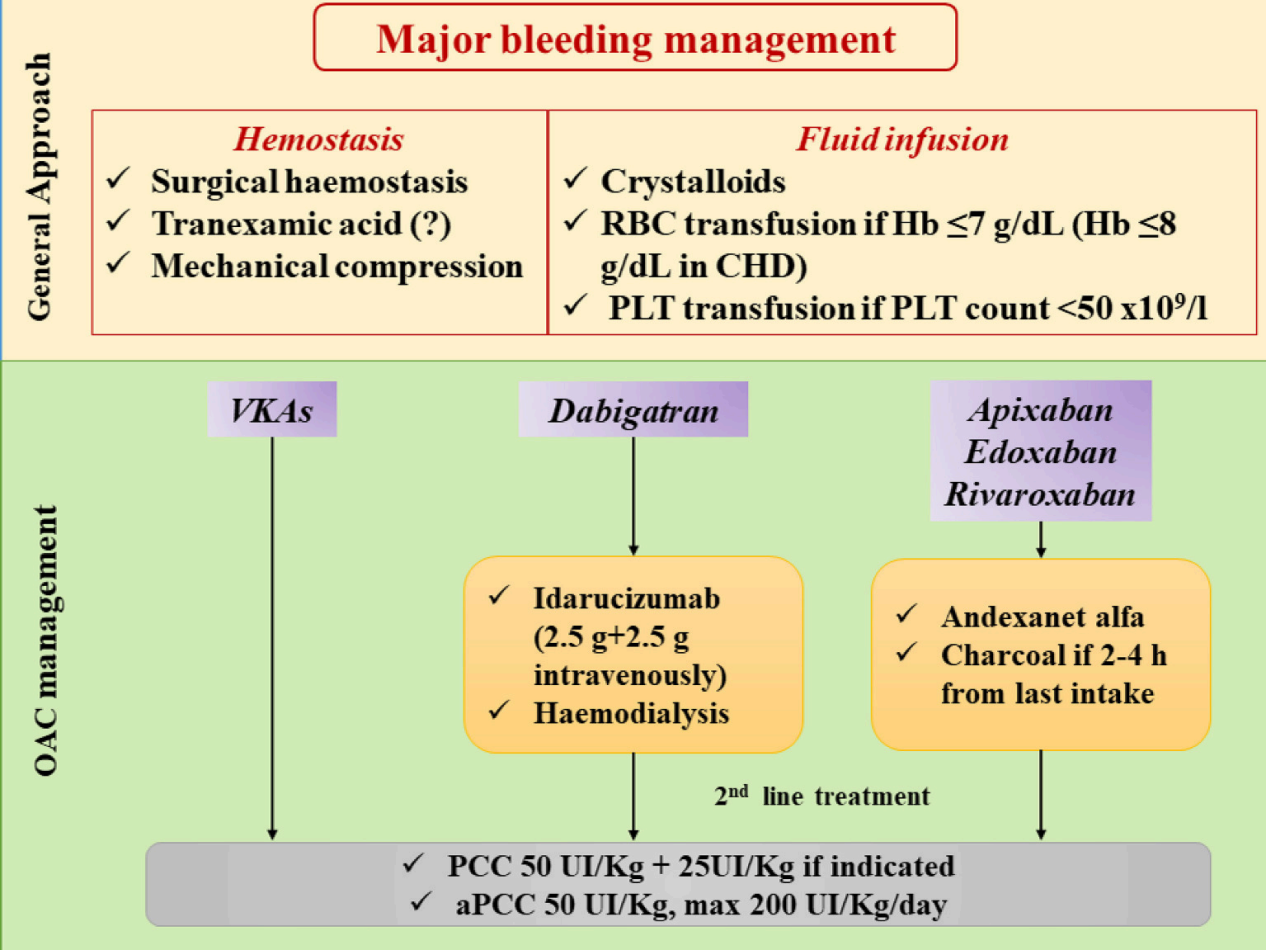
Management of major bleeding in patients treated with different oral anticoagulants. CHD: coronay heart disease; Hb: hemoglobin; OAC: oral anticoagulation; PCC: prothrombin complex concentrate; PLT: platelets; RBC: red blood cell; VKAs: vitamin K antagonists.

Hemodynamic support and safe hemostasis should be obtained in all patients presenting with active bleeding (Figure 3). Information on time of last NOAC intake and eventually errors in the intake of pills should be obtained. This will help determine if the use of reversal agents or prothrombin complex concentrates (PCC) is required.

Two reversal agents for NOACs have been approved so far, namely Idarucizumab, which is a high affinity antibody fragment that inactivates Dabigatran within few minutes of bolus injection (40, 63, 64), and Andexanet alfa, a recombinant modified human factor Xa decoy protein studied as a reversal of inhibitor Xa factor drugs (65).

Furthermore, in patients with recent last intake of NOAC (2–4 h), charcoal administration and/or gastric lavage will reduce further exposure, and dialysis may be considered to clear Dabigatran (Figure 3).

The second line of treatment is the use of PCC of activated prothrombin complex concentrates (aPCC) (40, 63, 66). PCC and aPCC can be used as first-line agents when a specific reversal is not available.

Figure 3 shows the management of major bleeding in AF patient in NOAC therapy.

### Ischemic Stroke

In patients presenting with acute ischemic stroke, thrombolysis is recommended within 4.5 h from the onset of symptoms for a better outcome (67, 68), but it cannot be administered in patients with INR > 1.7 in VKAs or within 24 h from the last dose of NOAC. In selected cases, reversal agents could be used to proceed with thrombolysis.

A measure of plasma concentration of NOACs could be useful up to 4 h from the last intake of drugs, and if NOACs concentration is <30 ng/ml thrombolysis could be considered (40).

For patients who suffered a stroke during both optimal and suboptimal anticoagulation with VKAs, a switch to NOAC is recommended. Conversely, there is no firm evidence on the utility of switching to another NOAC after a cerebrovascular ischemic event, even if it is generally done in clinical practice.

Regarding the timing of re-initiation of OAC therapy after acute phase of ischemic stroke, risk of recurrent stroke and hemorrhagic infarction should be evaluated.

**Figure 4:**
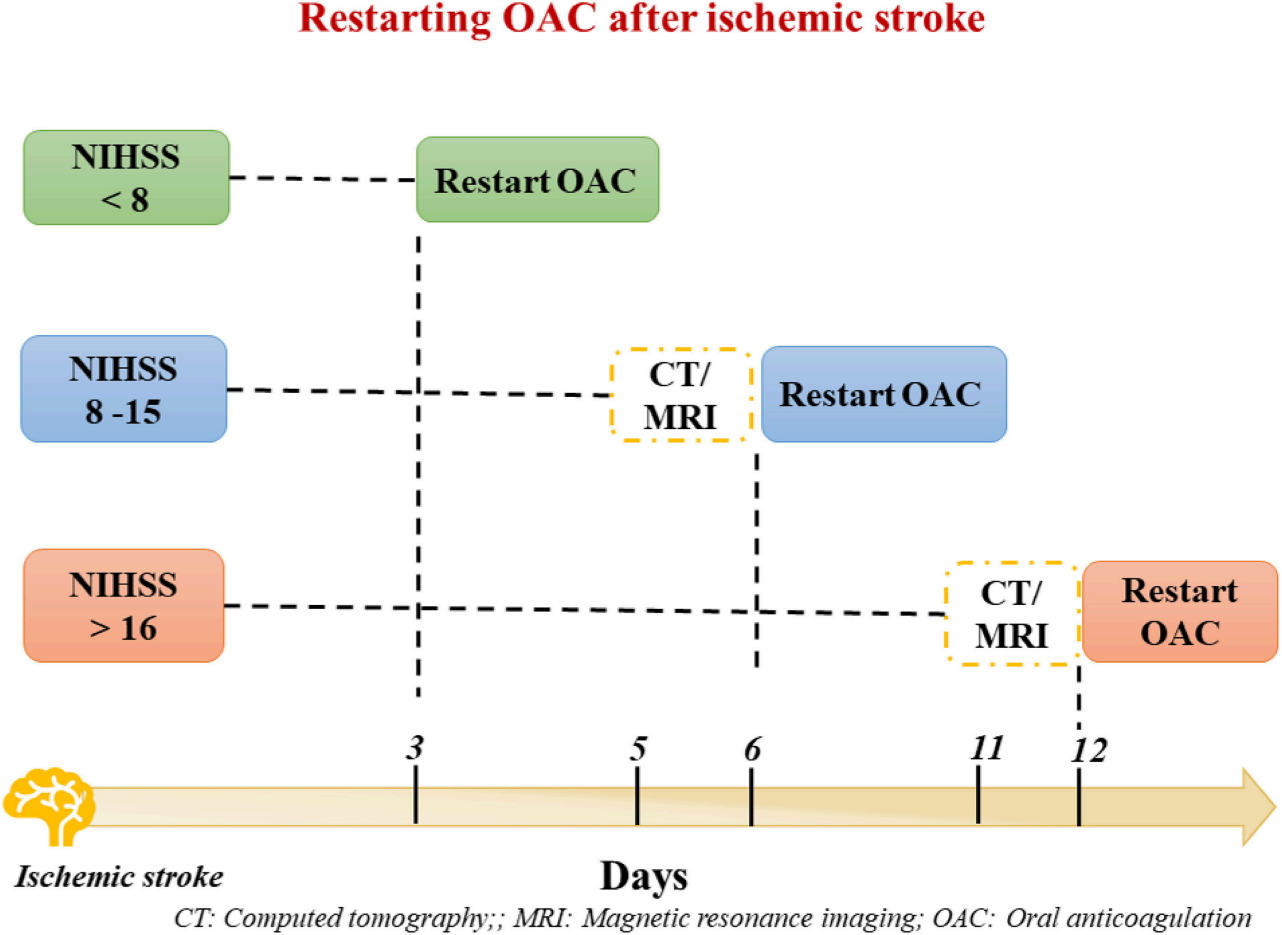
Scheme of restarting oral anticoagulation after ischemic stroke.

According to ESC guidelines recommendations (24) the National Institutes of Health Stroke Scale (NIHSS scale) should be used to evaluate the stroke severity, along with brain imaging (Figure 4). Thus, NOACs can be restarted ≥3 days in patients with mild, ≥6–8 days with moderate and ≥ 12–14 days with severe stroke size (Figure 4) (40). Conversely, NOACs could be continued in patients suffering a TIA.

### Ischemic Heart Disease

The risk of MI seems to be lower in AF patients treated with NOACs as compared to those on VKAs therapy. A recent study including 31,739 patients showed that the standardized 1-year risk of MI for patients on VKAs was 1.6% (95%CI 1.3–1.8), 1.2% (95%CI 0.9–1.4) for those on apixaban, 1.2% (95%CI 1.0–1.5) for those on dabigatran, and 1.1% (95%CI 0.8–1.3) for those on rivaroxaban (69).

Management of patients with AF and ischemic heart disease has been recently changed by results from randomized clinical trials with NOACs in patients undergoing percutaneous coronary intervention (PCI) due to an acute coronary syndrome (ACS) or to an elective procedure.

In the REDUAL-PCI Trial (70) (Randomized Evaluation of Dual Antithrombotic Therapy With Dabigatran vs. Triple Therapy With Warfarin in Patients with Non-valvular Atrial Fibrillation Undergoing Percutaneous Coronary Intervention), combination therapy of Dabigatran with a P2Y_12_ inhibitor was associated to a lower rate of major bleeding for both 110 and 150 mg bid, and a reduction in ICH for 150 mg bid compared with the TAT warfarin + P2Y_12_ inhibitor + aspirin, without increasing the risk of MI and stent thrombosis.

Also in the PIONEER AF-PCI (71) (Open-Label, Randomized, Controlled, Multicenter Study Exploring Two Treatment Strategies of Rivaroxaban and a Dose-Adjusted Oral Vitamin K Antagonist Treatment Strategy in Subjects with Atrial Fibrillation Who Undergo Percutaneous Coronary Intervention) trial, DAT with rivaroxaban 15 mg ad + single antiplatelet or rivaroxaban 2.5 mg od + double antiplatelet showed a similar efficacy compared to the TAT with warfarin.

Recent guidelines incorporated evidence from these trials, and suggested combination therapy with NOACs as a safer option over VKAs. In particular, the 2018 ESC guidelines on myocardial infarction (72) and the 2019 AHA focused update (21) recommend that in case of elective interventions or non-ST-elevation Myocardial infraction (NSTEMI), NOACs should be temporary discontinued making sure that catheter procedure is performed at least 12–24 h after last NOAC intake, and bridging therapy with LMWH (Fondaparinux or Enoxaparin) should be prescribed.

In patients presenting with ACS, a low dose Aspirin (150–300 mg) as well as a P2Y_12_ inhibitor should be added to NOAC, especially in case of ST-elevation Myocardial Infraction (STEMI). A primary PCI via a radial approach is recommended over fibrinolysis (73) and additional parenteral anticoagulation (UFH or LMWH) is needed regardless of the timing of last NOAC intake.

**Figure 5:**
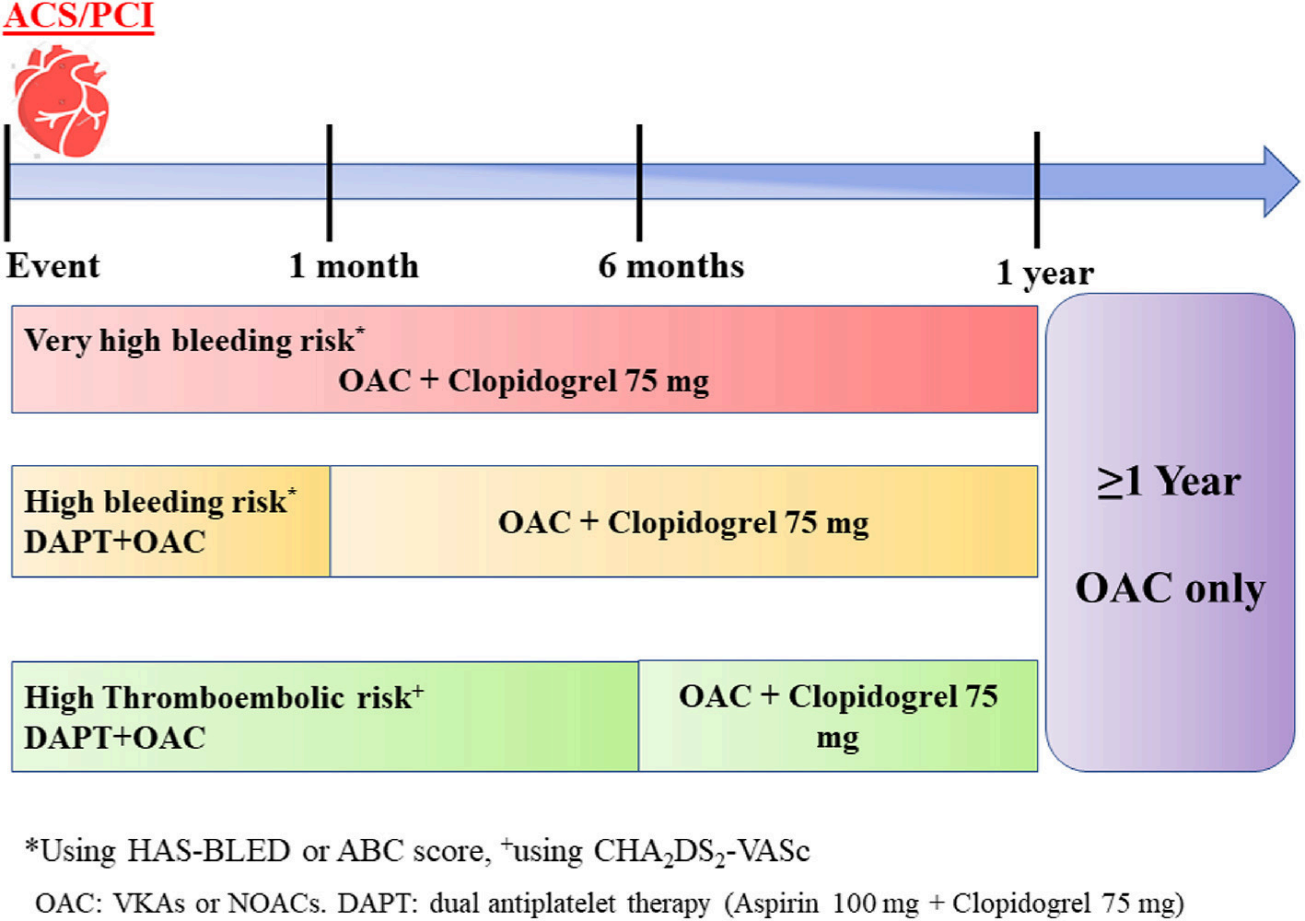
A simple scheme of antithrombotic therapy in patients with acute coronary syndrome (ACS)/percutaneous coronary intervention (PCI).

After revascularization procedure, patients should restart OAC as soon as parenteral anticoagulation is discontinued (Figure 5).

Triple antithrombotic therapy (TAT) including OAC (NOAC or VKA) in addition to two antiplatelet drugs (aspirin and a P2Y_12_ inhibitor) is necessary to prevent early stent thrombosis (40). However, given that TAT increases the risk of bleeding by 2 to 3-fold, the duration of TAT should be individualized depending on bleeding and ischemic risk (74) (Figure 5).

Thus, in patients classified as very-high risk of bleeding (Figure 5), TAT should be avoided and dual antithrombotic therapy (DAT) with a NOAC plus P2Y_12_ inhibitor should be continued for 12 months and afterwards stepped down to OAC in monotherapy.

In case of a high bleeding risk, TAT should be given for 1 month and replaced by DAT until 12 months. Finally, if stroke risk is high, a TAT can be prolonged up to 6 months, followed by DAT for other 6 months.

The 2018 EHRA recommendations (40) and 2019 AHA guidelines suggest that NOACs are a safe alternative over VKAs in association to antiplatelet therapy.

In particular, dabigatran 110 mg bis in die (bid), apixaban 5 mg bid or edoxaban 60 mg once daily (od) could be considered as part of the TAT (28). As an alternative to TAT, a DAT regimen containing dabigatran 150 mg (or dabigatran 110 mg bid when dose reduction criteria are present) or Rivaroxaban 15 mg od plus P2Y_12_ inhibitor (clopidogrel) may be considered to reduce the risk of bleeding (21, 28).

Given the lack of data, the use of reduced dose apixaban and edoxaban in the PCI setting are based on their approved labels. After withdrawal of antiplatelet drugs after 6–12 months from the index event, apixaban 5 mg bid and edoxaban 60 mg od could be used. Regarding the decision on whether or not to increase Dabigatran 110 mg to 150 mg bid is at physician discretion, based on the individual risk of stroke and bleeding.

After 12 months from the ACS/PCI, OAC therapy alone is indicated in most patients with AF.

## Conclusions

We have now several tools to stratify the risk of ischemic and bleeding events in patients with AF, but the use of these scores should be always accompanied by a careful evaluation of individual clinical risk factors, especially those potentially modifiable.

Clinicians should be aware of how to manage antithrombotic therapies in different clinical settings, the most challenging being represented by acute ischemic cardiac or cerebrovascular disease.

Despite the use of NOACs has significantly reduced the risk of major bleeding such as the ICH, their use in specific settings, such as in patients with advanced chronic kidney disease and in patients needing a combination therapy with antiplatelet drugs, is still an evolving clinical scenario.

A pro-active integrated approach to patients with AF is the mainstream to reduce not only thromboembolism but also cardiovascular disease in this patient population.

## Author Contributions

DP, DM, RG, PP, and FV all contributed to the writing and revision of the manuscript.

### Conflict of Interest Statement

The authors declare that the research was conducted in the absence of any commercial or financial relationships that could be construed as a potential conflict of interest.
